# Scheduling in Sensor Grid Middleware for Telemedicine Using ABC Algorithm

**DOI:** 10.1155/2014/592342

**Published:** 2014-12-03

**Authors:** T. Vigneswari, M. A. Maluk Mohamed

**Affiliations:** ^1^Kings College of Engineering, Punalkulam, Tamilnadu 613303, India; ^2^System Software Group, M.A.M College of Engineering, Tiruchirappalli, Tamilnadu 621105, India

## Abstract

Advances in microelectromechanical systems (MEMS) and nanotechnology have enabled design of low power wireless sensor nodes capable of sensing different vital signs in our body. These nodes can communicate with each other to aggregate data and transmit vital parameters to a base station (BS). The data collected in the base station can be used to monitor health in real time. The patient wearing sensors may be mobile leading to aggregation of data from different BS for processing. Processing real time data is compute-intensive and telemedicine facilities may not have appropriate hardware to process the real time data effectively. To overcome this, sensor grid has been proposed in literature wherein sensor data is integrated to the grid for processing. This work proposes a scheduling algorithm to efficiently process telemedicine data in the grid. The proposed algorithm uses the popular swarm intelligence algorithm for scheduling to overcome the NP complete problem of grid scheduling. Results compared with other heuristic scheduling algorithms show the effectiveness of the proposed algorithm.

## 1. Introduction

Telemedicine plays a very important role in patient management and has been effectively used for intrahospital transport of patients. Live monitoring of patients across hospitals creates new challenges. Similar issues arise as where to process the data captured in real time. Sensor grid can overcome some of the challenges faced in telemedicine.

Human life can be made more comprehensive by equipping it with modern diagnosis which utilizes the advancement in the field of information and communication and this arena is termed as telemedicine. Sensor grid is one among the computing methods which can be used efficiently to provide such a service. Grid computing [1, 2] is a growing distributed computing paradigm which enables coordinated sharing of heterogeneous resources across the globe. Sensor networks have [3] group of sensor nodes that are deployed to receive real time values about the parameters which they sense. Sensor grid [4] is the integration of sensor network and grid computing by which both of the paradigms can complement each other with their own strengths.

The advantages of merging sensor network and grid computing to form sensor grids [5, 6] include the following.

- Large amount of real time data generated by sensors can be processed and stored in the grid.
- Set of sensors can be shared by different user based on the application they are using.
- Pervasive seamless access to sensor data is made possible.

A sensor grid architecture to monitor patients who have undergone transplantation surgery is proposed in our previous work which includes the architecture for the sensor middleware [7, 8]. Scheduling component is a significant constituent in the proposed middleware. In general, scheduling discovers resources and allocates suitable tasks on appropriate resource to meet the requirement of the job handled. The major criteria for a good scheduling algorithm are response time, optimized resource utilization, load balancing, and meeting QoS constraints.

The scheduler in grid assigns jobs to the resources in an optimum way. Jobs arrive at the grid environment specifying the requirement about the resources. The assignment of jobs to the resources should be optimal to minimize the makespan, minimize the cost of allocated resources, and maximize the throughput [9]. Grid monitoring [10] collects the status and performance details of a large-scale distribution system. The parameters such as load on the system, number of jobs in the running state, and the performance of each job in running state are gathered to notify the behavior of the grid environment to the consumers.

Various scheduling algorithms have been proposed in literature for the grid environment. All algorithms fall into the taxonomy proposed in [11]. The first level in taxonomy is local versus global scheduling algorithms. In local scheduling, scheduler schedules the processes available on a single CPU. Global scheduler allocates processes to multiple CPUs. Grid scheduling uses global scheduling. Global scheduling uses static and dynamic scheduling. In static scheduling, the information about the jobs and resources are available at the time of scheduling. In dynamic scheduling, it is impossible to predict the arrival time and resource request of jobs, earlier to the time of scheduling. First in first out (FIFO), balance constrained techniques, and cost constrained techniques are some of the techniques which have been used in grids [12].

Due to the NP completeness of grid scheduling, metaheuristics techniques like particle swarm optimization (PSO), ant colony optimization (ACO), genetic algorithm (GA), and artificial bee colony (ABC) have been used effectively for grid scheduling [13–16].

This paper proposes ABC algorithm that can be used in scheduling of resource for the middleware discussed in [8] to provide a scheduling solution which optimizes the makespan of the submitted task.

## 2. Related Works

Telemedicine architecture using a sensor with P2P overlay has been proposed and a middleware has been developed for the abovementioned architecture. Among the middleware components, scheduling plays an inevitable role such that a proper scheduling may save a life. The data sets from the sensor should be scheduled to a computational resource for execution in the SaaS. The SaaS may send alert if the vital signs are abnormal and doctor reviews the patient immediately. An analysis of execution times of scheduling algorithms in [17] shows that taboo search algorithm is a suitable choice for applications including telemedicine involving static scheduling. The creation time is relatively small and simultaneously average execution time of the schedule is minimal. The total processor cycle consumption model [18] has shown to be useful for independent and coarse-grain task scheduling, that is, scheduling in which the computation time in grid nodes is superior to data transmission time.

A resource-performance-fluctuation-aware workflow scheduling algorithm proposed in [19] considers dynamic resource performance fluctuation in the grid, and scheduling is performed according to its knowledge of the fluctuation. This new algorithm works in an offline way which allows it to be easily set up and run with lower cost. The variation in the computing capability of heterogeneous nodes is also reflected on our proposed algorithm. A scheduling algorithm based on PSO [20] is proposed for task scheduling problem on computational grids. The proposed system reduced minimum completion time (MCT). In [21], adaptive workload balancing algorithm (AWLB) was incorporated into the distributed analysis environment (DIANE) user-level scheduling (ULS) environment. This gives the capability to select resources by the AWLB which is most suitable for the application, and the ULS environment is equipped with an advanced strategy to optimize resource usage. Optimization of the workload performed by the AWLB is adaptable to the resource characteristics (CPU power, memory, network bandwidth, input/output (I/O) speed, etc.) and to the corresponding application requirements.

Two models to predict job completion time in a service grid were proposed by Gao et al. [22]. The single service model predicts job completion time in a grid providing only one service type. Multiple services model predicts job completion time in a grid offering multiple types of services. To confront new challenges in grid environment job scheduling, Fidanova and Durchova [23] presented a heuristic scheduling algorithm designed to achieve high throughput computing in a grid environment. The authors proposed ACO which guarantees load balancing.

Fidanova [24] introduced a grid computing tasks scheduling algorithm based on simulated annealing (SA). Tasks are first collected in a set and then scheduled. So task arrival time is unimportant. Tasks scheduled on the same machine form a local queue related to the machine. Tasks queue is sent on to the machine when running tasks from a previous queue. Thus, sending time does not influence makespan time.

Scheduling workflows problem regarding quality of service (QoS) requirements is challenging. Though there are algorithms for grid workflow scheduling, most tackle problems with a single QoS parameter or with smaller workflows. An ACO algorithm to schedule large-scale workflows with various QoS parameters was proposed by Chen and Zhang [25] which enabled users to specify their QoS preferences and also define minimum QoS thresholds for specific applications. The algorithm's objective is to find a solution meeting all QoS constraints and optimizing the user-preferred QoS parameter. Based on workflow scheduling characteristics, 7 new heuristics for ACO approach were designed and an adaptive scheme that allowed artificial ants to select pheromone value based heuristics was proposed. Experiments done on ten workflow applications with 120 tasks demonstrate the proposed algorithm's effectiveness.

Di Martino and Mililotti [26] designed a two-level scheduling system, with the first level being formed by a computing nodes set (the sites participating in the grid)—each with a local scheduling policy—and a second level formed by super scheduler. The local scheduler accepts one job at a time and allocates it on local hardware regarding current (local) information. Results of grid jobs allocation simulation were presented. Garg and Singh [27] proposed the design/implementation of hierarchical discrete particle swarm optimization (H-DPSO) for grid environment's dependent task scheduling. In HDPSO, particles are dynamic, hierarchically arranged with good particles lying above and having much influence on swarm. The biobjective version of the problem is to minimize makespan and total cost simultaneously when optimization criteria were considered. The H-DPSO based scheduler was evaluated through various application task graphs. Simulation analysis shows that H-DPSO based scheduling for grid computing is highly viable and effective.

A binary artificial bee colony (BABC) algorithm for grid computing scheduling was proposed by Kim et al. [28]. An efficient binary artificial bee colony extension of BABC that incorporates a flexible ranking strategy (FRS) to improve balance between exploration and exploitation was proposed. The FRS generates and uses new solutions for diversified search in early generations and speeds up convergence in latter generations. Two variants to minimize the makespan were introduced. A fixed number of best solutions are employed with FRS in the first, while in the second the number of best solutions is reduced with every new generation. Simulation results for benchmark job scheduling issues reveal that the proposed method's performance is better than alternatives like SA, GA, and PSO.

Khanli et al. [29] presented a reliable job scheduler using resource fault occurrence history (RFOH) in grid computing. To improve the reliability, RFOH stored the number of faults occurring and number of jobs in execution. Based on the RFOH information genetic algorithm (GA) was used to find an optimum schedule. Pooranian et al. presented GLOA (a new job scheduling algorithm for grid computing) [30]. Group leader optimization algorithm (GLOA) is an optimization algorithm inspired by the role of leaders in a social group. The problem space was separated into multiple small parts, and each part was processed separately to find an optimal solution in parallel. GLOA was used to find an optimum schedule for the arrival of jobs with available resources. GLOA reached optimal solution quickly. Result showed that makespan was lower when comparingto all other traditional scheduling algorithms.

Karimi and Motameni presented tasks scheduling in computational grid using a hybrid discrete particle swarm optimization (HDPSO) [31]. The initial solution was obtained by Min-Min algorithm. For each job, the set of minimum completion time on all the available processors were found. Then, for each task, processor was selected which gave minimum expected completion time. Simulations were conducted with a specific number of jobs, and the processing time for each processor was preassumed. Makespan and throughput were taken for evaluating the performance of the scheduling. Results were compared with algorithms such as Min-Min and Max-Min algorithms. Comparison showed that HDPSO algorithm gave minimum makespan and maximum throughput. Mandloi and Gupta presented adaptive job scheduling for computational grid based on ACO with genetic parameter selection [32].

Selvi and Umarani presented comparative study of GA and ABC for job scheduling [33]. For efficient job scheduling in a grid environment, functions similar to genetic operations and bees behaviour were combined. Numerical results showed that hybrid GA-ABC job scheduling gave high accuracy and efficiency with minimum job completion time when comparing to GA and ABC scheduling algorithms.

Apart from these generic scheduling algorithms, some algorithms in context with telemedicine are also investigated. A sensor schedule service [34] injects the sensors in the targets based on the request from user. The sensor schedule is designed in a way such that it provides availability, fidelity, and QoS along with economy. Service-oriented grid [35] has been developed by GEMSS project to support the provision of medical simulation services by service providers to clients such as hospitals. It uses queue scheduling with advanced reservation for allocating resources. Arogyasree [36] introduced a context-aware scheduling which schedules patients to appropriate doctor based on the ailment they can handle with lesser response time. A grid middleware [37] that practices event based communication in in vivo sensor nodes consists of a lightweight rendezvous algorithm. This algorithm schedules temperature for in vivo sensor nodes deployed for joint operation of hyperthermia, radiotherapy, and chemotherapy.

## 3. Structural Overview

**Figure 1:**
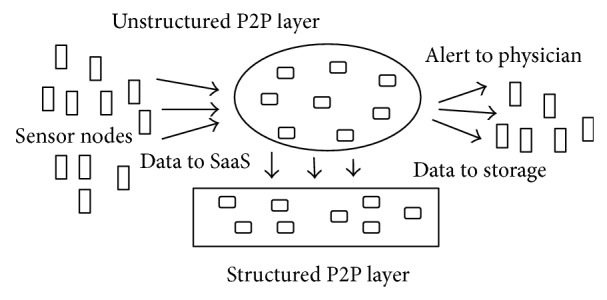
Structural overview.

Sensors attached to the patient's body send the vital sign values to the grid through a mobile device. The grid is designed as a two-layered P2P architecture to avoid centralized control. The peer to peer arrangement of grid nodes also improves scalability and fault tolerance. The first layer is the structured P2P layer used for analyzing the vital sign parameters. An application to analyze the values is made available in the grid nodes as SaaS. During the analysis if the SaaS finds that the parameter values exceed beyond normal values, an alert message is sent to the physician. The physician advises proper medication accordingly. The sensors are also abstracted as objects and stored in this layer. The second layer is a structured P2P layer for storing the vital parameter values. The patient or physician can retrieve the data from the database to know the history of a particular patient. This data can be shared with proper access rights. Distributed pipe communication is used for all the communications that take place between the sensors and grid nodes. The architectural overview is shown in Figure 1.

**Figure 2:**
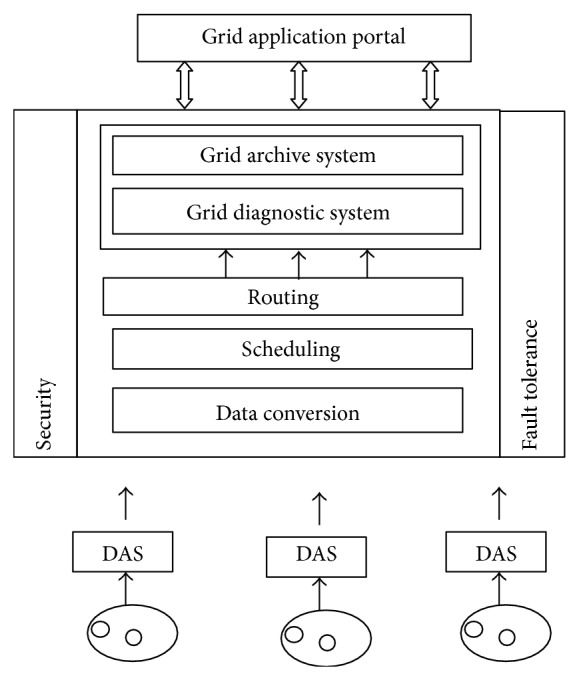
Middleware architecture.

The proposed middleware is shown in Figure 2 and has been designed to achieve the scope of the above given architecture. The sensor data is sent to a data acquisition system (DAS) which acts as a mobile base station. Then a data conversion element in the middleware converts into xml format so that it can be used in grid. The scheduling component allocates computational resource for analyzing these data sets in the SaaS. The nodes participating in the grid are grouped into zones based on their locality. The grid archive system stores the patient history in a database with timestamp for continuous monitoring.

## 4. Scheduling Algorithm

The middleware proposed by us aids in continuous monitoring of patients and sends alerts to the doctors if the patients vital sign values are abnormal. The data sets from DAS should be scheduled to a computational resource to analyze the data. The scheduling component should efficiently use the resources. In this regard, we have adapted ABC for resource scheduling.

### 4.1. Methodology

Artificial bee colony algorithm is based on the social behavior of honey bee colonies, and it can be applied to several optimization problems [14]. Honey bees share information about the location, quantity, and quality of foods. This information sharing activity can be used for resource management problems of grid scheduling. There are three types of honey bees in a bee colony. They are onlooker bees, employed bees, and scout bees. Employed bees search the locations of food in parallel and inform other bees by dancing. Onlooker bees evaluate and select the best solution among the solutions given by all the employed bees. Scout bees start a new search for solution [38].

### 4.2. Artificial Bee Colony Algorithm (ABC)

A bee colony is considered a swarm with bees as individual agents. Each low-level component bee works through a global level swarm component to form a system. Thus, system's global behaviour is determined from individual's local behaviour where interactions/coordination among individuals lead to an organized system characterized by interacting collective behaviour through labour division, distributed simultaneous task performance, self-organization, and specialized individuals. Information exchange among bees results in formation of tuned collective knowledge [39].

ABC is designed to handle numerical optimization problems [40]. It is based in two natural processes: recruitment of bees to a food source and source abandonment. The difference between ABC and other swarm intelligence algorithms is that, in the former, problem's solutions are represented by food sources, not bees. In comparison, bees act as variation operators discovering new food sources based on existing ones. Three bee types are considered in ABC: employed, onlooker, and scout bees. The employed bees are equal in number to the number of food sources with an employed bee being assigned to each food source. On reaching the source, the bee calculates a new solution and retains the best solution (using greedy heuristics). When a source fails to improve after many iterations, it is dumped and replaced by food source located by a scout bee, which in turn involves a random calculation of a new solution.

### 4.3. The Procedure of ABC

Classical ABC includes 4 phases [41].

*Initialization Phase.* Food sources, with SN population size, are generated randomly by scout bees. The artificial bee number is NP. Each food source *x*_*m*_ is a vector to optimization problem, *x*_*m*_ has *D* variables, and *D* is searching space dimension of objective function needing optimization. Initiation food sources are produced randomly by

(1) $$\begin{array}{c} x_{m}=l_{i}+\text{rand}\left(0,1\right)*\left(u_{i}-l_{i}\right), \end{array}$$

where *u*_*i*_ and *l*_*i*_ are upper and lower bound of the objective function's solution space and rand⁡(0,1) is a random number within the range [0,1]. 

*Employed Bee Phase.* Employed bees fly and locate a new food source in the food source's neighbourhood. A high quantity food source is selected. A neighbour food source *v*_*mi*_ is determined/calculated by

(2) $$\begin{array}{c} v_{mi}=x_{mi}+\phi_{mi}\left(x_{mi}-x_{ki}\right), \end{array}$$

where *x*_*k*_ is randomly selected food source, *i* is randomly chosen parameter index, and *ϕ*_*mi*_ is a random number within range [−1,1]. The food source fitness is essential to find a global optimal. In ABC, fitness is computed using (3), after which a greedy selection is applied between *x*_*m*_ and *v*_*m*_:

(3) $$\begin{array}{c} \text{fi}{\text{t}}_{m}\left(x_{m}\right)=\left\{\begin{array}{c} \frac{1}{1+f_{m}\left(x_{m}\right)},f_{m}\left(x_{m}\right)>0\\ 1+\left|f_{m}\left(x_{m}\right),f_{m}\left(x_{m}\right)<0\right| \end{array}\right\}, \end{array}$$

where *f*_*m*_(*x*_*m*_) is the objective function value of *x*_*m*_. In this work, makespan and available bandwidth based objective function were used. 

*Onlooker Bee Phase.* Onlooker bees see waggle dance in dance area and calculate food sources profitability and randomly choose a higher food source. Food source quantity is evaluated by profitability (*P*_*m*_) of all food sources. *P*_*m*_ is determined by

(4) $$\begin{array}{c} P_{m}=\frac{\text{fi}{\text{t}}_{m}\left(x_{m}\right)}{\sum_{m-1}^{\text{SN}}\text{fi}{\text{t}}_{m}\left(x_{m}\right)}, \end{array}$$

where fit_*m*_(*x*_*m*_) is the fitness of *x*_*m*_. The objective function and fitness function used in this work are the same. 

*Scout Phase.* The scouts randomly search for new solutions. If solution *x*_*i*_ is abandoned, a new solution *x*_*m*_ is discovered. *x*_*m*_ is defined by

(5) $$\begin{array}{c} x_{m}=l_{i}+\text{rand}\left(0,1\right)*\left(u_{i}-l_{i}\right), \end{array}$$

where *x*_*m*_ is new generated food source, rand⁡(0,1) is a random number within range [0,1], and *u*_*i*_ and *l*_*i*_ are upper and lower bound of objective function's solution space.

**Figure 3:**
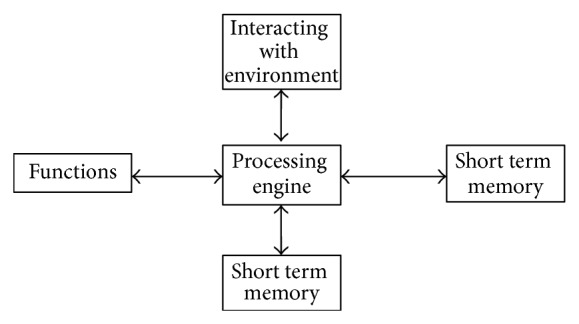
Architecture of artificial bee colony system.

The architecture of an ABC system is shown in Figure 3.

In the algorithm's first step, ${\vec{x}}_{i}$ (*i* = 1,…, SN) solutions are randomly produced where SN is total number of food sources. In the algorithm's second step, for every employed bee, whose total number equals half the number of food sources, a new source is produced through (2). In the algorithm's third step, an onlooker bee chooses a food source with probability given by (4) and produces a new source in chosen food source site by (2). Onlookers are distributed to good food sources only, thus eliminating suboptimal solutions. If number of iterations by which a source cannot be improved is bigger than a predetermined limit, source is considered exhausted. Employed bee linked to exhausted source is now a scout searching randomly in the problem domain by

(6) $$\begin{array}{c} x_{ij}=x_{j}^{\min}+\left(x_{j}^{\max}-x_{j}^{\min}\right)*\text{rand.} \end{array}$$

**Figure 4:**
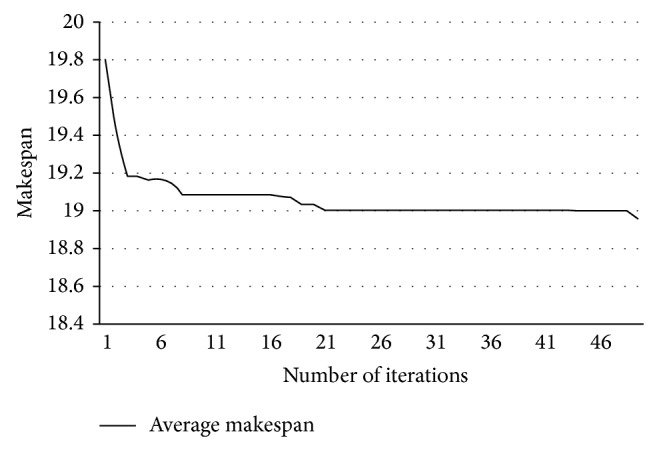
Average makespan.

**Figure 5:**
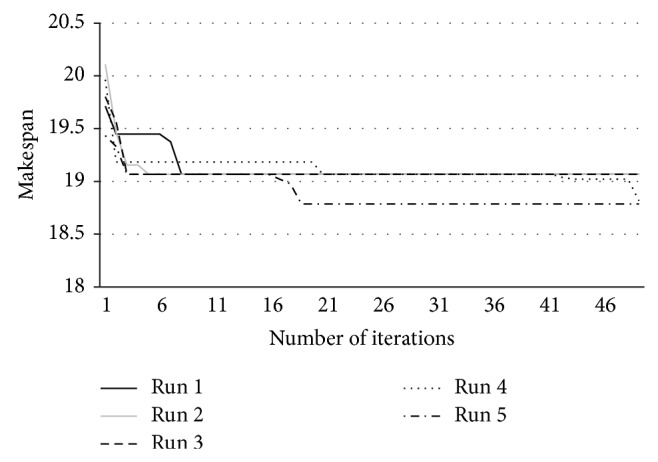
Makespan for all the 5 runs with 50 iterations.

Simulations are conducted with 25 jobs, and the resources are grouped into 5 clusters. Using dynamic arrival time of jobs with different requirement of resources, the proposed ABC scheduling algorithm is run five times. During every run, makespan value is recorded.  Figure 4 shows the obtained average makespan and Figure 5 shows the individual makespan obtained in each run.

Simulations were also conducted by varying the number of jobs from 25 to 175 in increments of 25 and also increasing the number of resources from five to fifteen. The proposed ABC is compared with Min-Min scheduling and ACO based scheduling.  Table 1 shows the average makespan obtained for different number of jobs and resources.

It is observed from the results that the makespan reduces significantly as the number of resources increases. The proposed ABC reduces makespan when compared to Min-Min scheduling and ACO scheduling in the ranges of 2.9% to 3.66% and 2.78% to 3.33%, respectively. Similarly, the proposed technique improves the makespan in the range of 2.89% to 3.98% compared to Min-Min scheduling and by 2.52% to 3.32% compared to ACO method when the number of resources is fifteen.

Table 2 shows the resource utilization.

From Table 2, it is seen that the resource utilization by proposed technique is higher. The proposed ABC improves resource utilization when compared to Min-Min scheduling in the range of 15.17% to 15.69% and by 1.16% to 1.73% when compared to ACO scheduling when the number of resources is 5.  Table 3 shows the standard deviation obtained across five runs.

It is observed from Table 3 that the standard deviation for the proposed ABC is significantly lower than that of Min-Min scheduling justifying the stability of the proposed algorithm. The inherent advantages of the ABC algorithm such as the algorithm requiring fewer control parameters and good balance between local and global search processes produce better results as observed from the simulation results.

## 5. Conclusion

A sensor grid middleware has been designed to receive vital sign from the patients and monitor them continuously to check for abnormality. The scheduling component of this middleware is an imperative element which schedules the data to computational resource for executing in a SaaS. In this paper, we have adapted ABC scheduling algorithm which will efficiently schedule the data sets to computational resource to meet out the scope of the middleware successfully. Simulations are conducted with different number of jobs and resources. From the obtained simulations, it can be observed that proposed ABC algorithm performs better than ACO and Min-Min scheduling.

## Figures and Tables

**Table 1:** Average makespan.

Number of jobs	Number of resources = 5			Number of resources = 15		
	Min-Min	ACO	ABC	Min-Min	ACO	ABC
25	19.42	19.34	18.81	7.02	7.02	6.82
75	59.78	59.82	57.86	21.48	21.41	20.71
125	101.24	101.09	98.29	34.62	34.12	33.27
175	142.64	141.98	137.52	49.54	49.55	48.09

**Table 2:** Resource utilization.

Number of jobs	Number of resources = 5			Number of resources = 15		
	Min-Min	ACO	ABC	Min-Min	ACO	ABC
25	68.64	81.06	80.12	70.27	74.87	73.34
75	69.75	82.26	81.2	70.51	80.28	78.75
125	70.09	83.33	81.9	71.57	80.34	78.75
175	70.11	83.11	82.05	72.17	81.13	79.76

**Table 3:** Standard deviation (±).

Number of jobs	Number of resources = 5			Number of resources = 15		
	Min-Min	ACO	ABC	Min-Min	ACO	ABC
25	0.83	0.7	0.68	0.89	0.82	0.81
75	0.73	0.42	0.42	0.79	0.88	0.87
125	0.66	0.45	0.45	0.7	0.33	0.33
175	0.75	0.46	0.45	0.41	0.46	0.46
